# Hepatology consultation is associated with decreased early return to alcohol use after discharge from an inpatient alcohol use disorder treatment program

**DOI:** 10.1097/HC9.0000000000000414

**Published:** 2024-04-12

**Authors:** Hanna L. Blaney, Mian B. Khalid, Alexander H. Yang, Bilal A. Asif, Anusha Vittal, Natasha Kamal, Elizabeth C. Wright, Tomilowo Abijo, Chris Koh, David George, David Goldman, Yvonne Horneffer, Nancy Diazgranados, Theo Heller

**Affiliations:** 1 Liver Diseases Branch, National Institute of Diabetes and Digestive and Kidney Diseases, National Institutes of Health, Bethesda, Maryland, USA; 2 Office of the Director, National Institutes of Diabetes and Digestive and Kidney Diseases, National Institutes of Health, Bethesda, Maryland, USA; 3 Office of the Clinical Director, National Institute on Alcohol Abuse and Alcoholism, National Institutes of Health, Bethesda, Maryland, USA

## Abstract

**Background:**

Alcohol cessation is the only intervention that both prevents and halts the progressions of alcohol-associated liver disease. The aim of this study was to assess the relationship between a return to alcohol use and consultation with hepatology in treatment-seeking patients with alcohol use disorder (AUD).

**Methods:**

Two hundred forty-two patients with AUD were enrolled in an inpatient treatment program, with hepatology consultation provided for 143 (59%) patients at the request of the primary team. Patients not seen by hepatology served as controls. The primary outcome was any alcohol use after discharge assessed using AUDIT-C at 26 weeks after discharge.

**Results:**

For the primary endpoint, AUDIT at week 26, 61% of the hepatology group and 28% of the controls completed the questionnaire (*p*=0.07). For the secondary endpoint at week 52, these numbers were 22% and 11% (*p* = 0.6). At week 26, 39 (45%) patients in the hepatology group versus 31 (70%) controls (*p* = 0.006) returned to alcohol use. Patients evaluated by hepatology had decreased rates of hazardous alcohol use compared to controls, with 36 (41%) versus 29 (66%) (*p* = 0.008) of the patients, respectively, reporting hazardous use. There were no significant differences in baseline characteristics between groups and no difference in rates of prescribing AUD therapy. There was no difference in outcomes at 52 weeks.

**Conclusions:**

Patients evaluated by hepatology had significantly lower rates of return to alcohol use and lower rates of hazardous drinking at 26 weeks but not at 52 weeks. These findings suggest that hepatology evaluation during inpatient treatment of AUD may lead to decreased rates of early return to alcohol use.

## Graphical abstract

**Figure:**
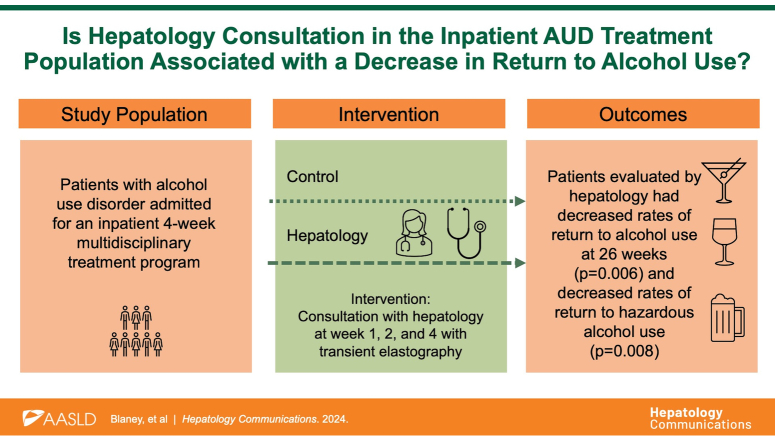

## INTRODUCTION

Alcohol-associated liver disease (ALD) is a common consequence of excessive alcohol use, with up to one-third of patients with alcohol use disorder (AUD) developing various forms of ALD, ranging from steatosis to cirrhosis and HCC.^1^,^2^ ALD is the leading cause of cirrhosis in many high-income countries. ALD has a dose-response relationship with the amount of alcohol consumed, with the steepest increase seen among women.^3^,^4^

ALD progresses silently and often remains undiagnosed until the patient becomes symptomatic or presents with decompensated stigmata of liver disease. Patients with ALD are rarely seen at the early stages of the disease.^5^ The odds of late diagnosis are 12 times higher for patients with ALD compared to those with viral hepatitis.^6^ Early identification of liver disease followed by reduction or cessation of alcohol is key to preventing the progression of ALD. Abstinence from alcohol has been shown to improve morbidity and mortality at all stages of ALD.^7^,^8^ Studies have demonstrated that pharmacotherapy for AUD reduces the incidence and progression of ALD and is associated with improved survival.^9^,^10^

Rates of return to drinking after treatment for AUD are high, with 60%–80% and 70%–80% of patients returning to drinking after 3 and 12 months, respectively.^11^ A few studies suggest that the knowledge of liver disease can influence drinking behavior.^12^,^13^,^14^,^15^ However, little is known about whether consultation with a hepatologist affects outcomes of AUD in treatment-seeking patients without known advanced liver disease.

The aim of this study was to assess the relationship between change in return to alcohol use and consultation with a hepatologist in treatment-seeking patients with AUD. The secondary aim was to evaluate if knowledge of liver disease in patients with evidence of liver disease influenced the return to drinking.

## METHODS

Patients seeking treatment for AUD from January 2017 to August 2022 were enrolled per protocol in a 4-week interdisciplinary inpatient treatment program at the National Institute on Alcohol Abuse and Alcoholism (NIAAA) at the National Institutes of Health (NIH) Clinical Center in Bethesda, Maryland. Inclusion criteria for this natural history protocol included adults aged 18 years or older and willingness to complete the study including willingness to undergo blood testing, genetic testing, and MRI. All patients underwent clinical evaluation and received multidisciplinary care under a primary psychiatry and addiction medicine team, including individual and group behavior counseling, complete history and physical examination, and social work evaluation. All patients were diagnosed with AUD using the Structured Clinical Interview for DSM-5, and performed baseline Alcohol Use Disorder Identification Test (AUDIT) and Lifetime Drinking History questionnaires with an extensive history of alcohol intake taken to include amount, duration, and frequency.

Laboratory values were collected on all patients at admission, weeks 2, and 3 per protocol. All patients had the ability to access these results through electronic medical records. After discharge, all patients were invited to submit AUDIT questionnaires at week 26 and week 52.

Beginning in 2018, the majority of these patients were seen by the hepatology service with vibration-controlled transient elastography (VCTE) (Echosens, FibroScan) performed at weeks 1, 2, and 4 with patients in a fasting state. At least 10 liver stiffness measurements (LSMs) were recorded for all patients, with IQR <30%. The first visit with hepatology included an assessment of alcohol drinking history and liver disease, with a discussion of baseline labs and VCTE, and how alcohol adversely affects the liver. Follow-up visits included VCTE and discussion of subsequent results. Patients were seen by 6 different gastroenterology and hepatology fellows under the supervision of an attending hepatologist. Standard scripts were not used in patient counseling. Instead, fellows used their clinical judgment. All patients, regardless of evidence of liver injury or ALD, received counseling on the importance of abstinence from alcohol. While the majority of patients attended all 3 visits with hepatology with fibroscan performed, a few patients missed either week 2 or week 4 visits with hepatology.

Patients with elevated week 4 LSM, defined as VCTE kilopascal (kPa) ≥7, were analyzed as an ALD subgroup. Week 4 VCTE was used instead of week 1 VCTE, given data on the immediate inflammatory effect that alcohol has on the liver and associated increased LSM.^16^ Given the high prevalence of patients with liver steatosis from heavy alcohol use and subsequent improvement in Controlled Attenuation Parameter with cessation in alcohol use,^17^ as well as the high prevalence of steatosis in the general population, LSM was used to define the population with ALD. As this treatment population is generally healthy without known liver disease and we were aiming to detect early ALD, we chose a threshold LSM of 7 kPa instead of 8 kPa, which is typically used in screening for elevated liver stiffness.

Patients enrolled in this treatment program before 2018 and patients who did not see hepatology (due to unavailability of hepatology consultation) served as controls. Pre-COVID was defined as patients seen before April 2020.

All patients were offered pharmacotherapy for AUD when clinically appropriate by the primary psychiatry team. Patients who accepted a prescription medication for AUD (including Food and Drug Administration–approved medications such as oral or intramuscular naltrexone, disulfiram, and acamprosate, as well as off-label medications including gabapentin, topiramate, or baclofen) on discharge were counted as treated.

AUDIT-C was used as a surrogate for return to alcohol use, with a nonzero answer to question 1 “How often do you have a drink containing alcohol?” used as a marker of return to any alcohol use after discharge. An AUDIT-C result of ≥3 for women and ≥4 for men was used as a surrogate for hazardous alcohol use.^18^,^19^,^20^

Abnormal laboratory values for the control group were identified using the reference ranges for our laboratory, as these are the values patients and other providers recognize as abnormal.

### Statistics

Descriptive statistics were used to compare the clinical and demographic characteristics of the control and hepatology groups and patients with baseline LSM <7 or ≥7 kPa. All patients who saw hepatology at least once were included in the hepatology group. Continuous data were summarized by mean and standard deviations. Discrete and categorical data were summarized with frequency (count) and percentage. *t* tests or the Wilcoxon rank sum test were used to test for significant differences in continuous variables between control and hepatology groups while chi-square or Fisher exact tests were employed to test for significant differences in categorical variables between control and hepatology groups. Ordinal logistic regression was used for the individual AUDIT questions. Similar tests were done for the hepatology groups based on LSM. All statistical analyses were done with a significant value of alpha = 0.05, and a *p*-value lower than 0.05 for a two-sided test was considered to be statistically significant. The SAS statistical software (SAS Institute) was used for all statistical analyses.

### Outcomes

The primary outcome was a return to any alcohol use after discharge based on AUDIT-C at week 26. Secondary outcomes were a return to alcohol use based on AUDIT-C at 52 weeks and a return to hazardous alcohol use at 26 and 52 weeks.

### Ethics

All research was conducted in accordance with both the Declarations of Helsinki and Istanbul. All study participants provided written informed consent under the NIAAA Natural History Protocol 14-AA-0181 and approved by the NIH Institutional Review Board.

## RESULTS

**FIGURE 1:**
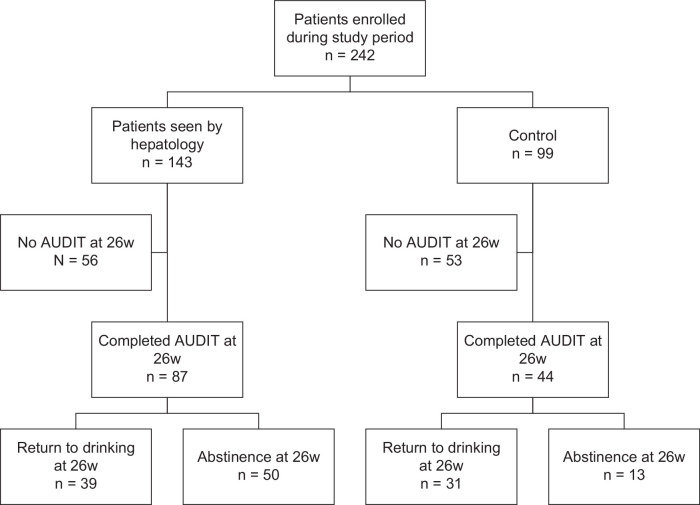
Study flowchart. Abbreviation: AUDIT, Alcohol Use Disorder Identification Test.

Two hundred forty-two patients were admitted during the study period, with 143 patients seen by hepatology. Of the hepatology group, 87 (61%) patients completed AUDIT at week 26 versus 44 (44%) of the controls (*p* = 0.01 by a simple chi-square test). This difference was no longer significant after adjusting for the year of admission (*p* = 0.7 by the Cochran-Mantel-Haenszel statistic) (Figure 1).

### Patient characteristics

There were no significant differences in baseline characteristics between groups including laboratory results, baseline AUDIT, and baseline drinking patterns, with the exception of the year of treatment enrollment and average drinks per day (Table 1). Patients in the hepatology group had higher incidences of both current and lifetime diagnoses of anxiety without comorbid post-traumatic stress disorder or obsessive-compulsive disorder (*p* = 0.03 and *p* = 0.04, respectively), with no significant differences in other current or lifetime mental health diagnoses. The mean age of both groups was 47 (SD: 11.6 and 11.1, *p* = 1). Of the hepatology group, 60/87 (69%) were male compared to 29/44 (66%) of controls (*p* = 0.7). About half identified as White, 44/87 (51%) of the hepatology group and 21/44 (48%) of the control group (*p* = 0.8). Most patients were seen pre-pandemic with 46/87 (53%) of the hepatology group and 32/44 (73%) control patients seen before April 2020 (*p* = 0.03). Patients in the hepatology group had significantly higher daily alcohol consumption, consuming 15.8 versus 12.5 drinks compared to controls (*p* = 0.02). There were no significant differences in rates of prescribing pharmacotherapy for AUD at discharge, with 51/87 (59%) in the hepatology and 21/44 (48%) in the control group receiving medication on discharge (*p* = 0.2). All patients in the control group who were prescribed pharmacotherapy received either naltrexone (88%) or naltrexone for extended-release injectable suspension (12%). Of the hepatology group, patients receive naltrexone (73%), naltrexone for extended-release injectable suspension (14%), or acamprosate (8%).

**TABLE 1:** Baseline characteristics—hepatology versus controls

	Hepatology		Control		
	N	Mean (SD) Median (IQR) N (%)	N	Mean (SD) Median (IQR) N (%)	*p*
Year of treatment^a^	87	2020.0 (1.16)	44	2019.0 (1.13)	<0.0001
Before April 2020^b^	87	46 (53)	44	32 (73)	0.029
Sex, male^b^	87	60 (69)	44	29 (66)	0.72
White race^b^	87	44 (51)	44	21 (48)	0.76
Age^a^	87	47.0 (11.6)	44	47.0 (11.1)	0.99
Frequencies of alcohol consumption					
Age at first drink^c^	87	14.3 (4.6) 15 (12–17)	43	15.0 (5.2) 15 (13–16)	0.43 0.79
Average drink per day^c^	87	15.8 (7.3) 15.2 (11.0–18.8)	42	12.5 (7.0) 10.9 (7.5–16.1)	0.017 0.0051
Drinks per 30 d^c^	87	384 (252) 339 (187–499)	42	330 (229) 268 (191–420)	0.24 0.21
Heavy-drinking days in last 90 d^c^	87	67.5 (26.1) 78 (45–90)	42	72.4 (23.5) 86 (48–90)	0.31 0.28
Heavy-drinking years^c^	86	17.2 (11.2) 14.5 (8.8–25)	43	14.7 (9.7) 13.0 (1.9–21.5)	0.21 0.29
Number of drinking days in last 90 d^c^	87	69.7 (24.3) 81 (56–90)	42	76.0 (20.9) 89 (72–90)	0.15 0.14
Total lifetime number of drinks (thousands) ^c^	86	74.9 (64.3) 60.4 (31.4–95.2)	43	65.6 (61.0) 50.5 (30.0–77.7)	0.43 0.32
Total lifetime drink^c^ (kg)	86	1049 (900) 846 (440–1333)	43	918 (854) 707 (420–1088)	0.43 0.32
Day 1 lab values					
Total bilirubin^a^	87	0.69 (0.90)	44	0.59 (0.49)	0.45
GGT^c^	87	178 (342) 77 (44–168)	44	202 (251) 82 (44–288)	0.65 0.57
MCV^a^	87	93.8 (5.94)	43	94.2 (7.03)	0.74
Platelet count^a^	86	226 (83.0)	43	215 (71.3)	0.45
ALT^c^	87	44.7 (35.4) 34 (23–57)	44	53.8 (55.1) 35 (21–62)	0.32 0.77
AST^c^	87	60.9 (52.0) 39 (24–82)	44	61.9 (52.8) 42 (24–48)	0.92 0.95
Day 21 lab values					
Total bilirubin^a^	83	0.45 (0.78)	27	0.39 (0.25)	0.55
GGT^c^	83	77.1 (94.2) 48 (30–79)	27	119 (125) 54 (40–168)	0.067 0.13
MCV^a^	75	94.0 (5.45)	23	95.3 (5.81)	0.33
Platelet count^a^	75	266 (78.0)	23	276 (93.4)	0.59
ALT^c^	83	29.1 (15.2) 34 (23–57)	26	68.9 (142) 32 (19–53)	0.17 0.11
AST^c^	83	26.1 (13.6) 39 (24–82)	26	48.7 (79.5) 25 (21–40)	0.16 0.13
Baseline AUDIT					
Q1. How often have a drink containing alcohol?	87	3.83 (0.58) 4 (4–4)	43	3.72 (0.80) 4 (4–4)	0.44 0.35
Q2. How many standard drinks a typical day?	87	3.38 (1.00) 4 (3–4)	43	3.02 (1.08) 3 (2–4)	0.066 0.033
Q3. How often ≥6 drinks on one occasion?	87	3.58 (0.77) 4 (3–4)	43	3.42 (0.82) 4 (3–4)	0.29 0.12
During the past year 6 mo					
Q4. How often not able to stop once started?	87	3.23 (1.19) 4 (3–4)	43	3.14 (1.19) 3 (3–4)	0.68 0.56
Q5. How often failed to do what was normally expected?	87	2.03 (1.31) 2 (1–3)	43	1.95 (1.27) 2 (1–3)	0.74 0.76
Q6. How often needed a drink in the morning?	87	2.41 (1.56) 3 (1–4)	43	2.56 (1.39) 3 (1–4)	0.61 0.76
Q7. How often had feelings of guilt or remorse?	87	2.51 (1.35) 3 (1–4)	43	2.54 (1.45) 3 (1–4)	0.91 0.78
Q8. How often unable to remember what night before?	87	1.79 (1.30) 2 (1–3)	43	1.77 (1.17) 2 (1–3)	0.91 0.87
Q9. Have you/someone else been injured result drinking	87	1.20 (1.68) 0 (0–2)	43	1.12 (1.59) 0 (0–2)	0.80 0.89
Q10. How often other concerned or suggested cut down?	87	3.59 (1.11) 4 (4–4)	43	3.58 (1.12) 4 (4–4)	0.98 0.98
AUDIT-C	87	10.8 (1.9) 12 (10–12)	43	10.2 (2.3) 11 (9–12)	0.10
AUDIT-D	87	7.7 (3.0) 8 (6–10)	43	7.7 (3.1) 8 (5–10)	0.96
AUDIT-H	87	9.1 (3.5) 9 (6–10)	43	9.0 (3.8) 9 (7–12)	0.91
AUDIT Total score	87	27.5 (6.4) 28 (23–33)	43	26.8 (7.5) 29 (24–32)	0.57
Other SUD current^b^	87	29 (33)	43	9 (21)	0.41
Other SUD lifetime^b^	87	59 (68)	44	29 (66)	0.83
Anxiety current^b^	87	30 (34)	44	10 (23)	0.17
Anxiety lifetime^b^	87	45 (52)	44	15 (34)	0.056
Anxiety, no PTSD or OCD current^b^	87	16 (18)	44	2 (5)	0.030
Anxiety, no PTSD or OCD lifetime^b^	87	21 (24)	44	4 (9)	0.039
Mood current^b^	87	17 (20)	44	13 (30)	0.20
Mood lifetime^b^	87	32 (37)	44	18 (41)	0.65
Depression current^b^	87	16 (18)	44	13 (30)	0.15
Depression lifetime^b^	87	31 (36)	44	17 (39)	0.74
PTSD current^b^	87	17 (20)	44	9 (20)	0.90
PTSD lifetime^b^	87	30 (34)	44	13 (30)	0.57
Pharmacotherapy for AUD					
Prescribed at discharge	87	51 (59)	44	21 (48)	0.24
LSM liver (kPA)					
LSM week 1 (kPa)	75	8.0 (10.9) 5.5 (3.9–7.7)	0	—	—
LSM week 2 (kPa)	81	7.3 (8.8) 4.9 (3.8–6.7)	0	—	—
LSM week 4 (kPa)	76	7.5 (9.4) 4.9 (4.0–6.1)	0	—	—

a *p* value is from a *t* test.

b *p* value is from a chi-square test.

c *p* values are from a *t* test and a Wilcoxon rank sum test.

*p* values are from a *t* test and ordinal logistic regression.

Abbreviations: ALT, alanine aminotransaminase; AST, aspartate aminotransferase; AUD, alcohol use disorder; AUDIT, Alcohol Use Disorder Identification Test; AUDIT-C, Alcohol Use Disorder Identification Test-Consumption; AUDIT-D, Alcohol Use Disorder Identification Test-Dependence; AUDIT-H, Alcohol Use Disorder Identification Test-Harm; GGT, gamma-glutamyl transferase; LSM, liver stiffness measurement; MCV, mean corpuscular volume; OCD, obsessive-compulsive disorder; PTSD, post-traumatic stress disorder; SUD, Substance Use Disorder.

### AUDIT week 26

At week 26, 39/87 (45%) patients in the hepatology group versus 31/44 (70%) in controls (*p* = 0.006) answered affirmatively to question 1 (AUDQ1) (>0 to “How often do you have a drink containing alcohol?”), with mean scores of 1.46 and 2.09, respectively (*p* = 0.05). Median AUDIT-C was 0 for the hepatology group and 4.5 for the control (*p* = 0.04), with mean scores of 3.56 for the hepatology group and 5 for the control (*p* = 0.09) (Table 2). Patients seen by hepatology also had decreased rates of hazardous alcohol use at week 26 as defined by AUDIT-C (>3 for females or >4 for males) compared to controls, with 36 (41%) versus 29 (66%) (*p* = 0.008) patients, respectively, reporting hazardous use.

**TABLE 2:** AUDIT at 26 and 52 weeks—hepatology versus control

	Hepatology		Control			
Variable	N	Mean (SD) Median (IQR) N (%)	N	Mean (SD) Median (IQR) N (%)	Difference means	*p*
Week 26 Audit						
Question 1^a^	87	1.46 (1.78) 0 (0–4)	44	2.09 (1.68) 2 (0–4)	−0.63	0.053 0.040
Question 1 >0^b^	87	39 (45)	44	31 (70)	−25%	0.0055
Question 2^a^	87	1.06 (1.50) 0 (0–2)	44	1.34 (1.41) 1 (0–2.5)	−0.28	0.30 0.12
Question 3^a^	87	1.05 (1.61) 0 (0–3)	44	1.57 (1.62) 1 (0–3)	−0.52	0.083 0.030
Question 4^a^	87	1.08 (1.67) 0 (0–3)	44	1.07 (1.50) 0 (0–2)	0.01	0.97 0.87
Question 5^a^	87	0.86 (1.45) 0 (0–2)	44	1.07 (1.58) 0 (0–2.5)	−0.21	0.46 0.34
Question 6^a^	87	1.07 (1.72) 0 (0–3)	44	1.11 (1.65) 0 (0–2.5)	−0.05	0.89 0.54
Question 7^a^	87	1.16 (1.68) 0 (0–3)	44	1.59 (1.68) 1 (0–3.5)	−0.43	0.17 0.074
Question 7 >0^b^	87	32 (37)	44	26 (59)	−22%	0.016
Question 8^a^	87	0.64 (1.19) 0 (0–1)	44	0.98 (1.41) 0 (0–1.5)	−0.33	0.16 0.13
Question 9^a^	87	0.39 (1.14) 0 (0–0)	44	0.46 (1.21) 0 (0–0)	−0.06	0.77 0.73
Question 10^a^	87	1.70 (1.92) 0 (0–4)	44	1.96 (1.95) 2 (0–4)	−0.25	0.48 0.48
AUDIT-C score^c^	87	3.56 (4.61) 0 (0–7)	44	5.00 (4.29) 4.5 (0–9)	−1.44	0.087 0.043
AUDIT-C ≥3 (F), ≥4 (M)^b^	87	36 (41)	44	29 (66)	−25%	0.0080
AUDIT-D score^c^	87	3.01 (4.45) 0 (0–7)	44	3.25 (4.22) 1 (0–6.5)	−0.24	0.77 0.34
AUDIT-H score^c^	87	3.90 (4.66) 2 (0–8)	44	4.98 (4.86) 4 (0–8)	−1.08	0.22 0.18
Total AUDIT score^c^	87	10.47 (13.08) 4 (0–24)	44	13.23 (12.22) 10 (1.5–24)	−2.76	0.25 0.089
Week 52 Audit						
Question 1^a^	40	1.78 (1.76) 1 (0–4)	17	2.18 (1.59) 2 (1–4)	−0.40	0.42 0.44
Question 1 >0^b^	40	24 (60)	17	13 (77)	−17%	0.23
Question 2^a^	40	1.38 (1.53) 1 (0–3)	17	1.59 (1.58) 1 (0–2)	−0.21	0.64 0.57
Question 3^a^	40	1.80 (1.80) 1.5 (0–4)	17	1.77 (1.52) 2 (0–3)	0.04	0.94 0.97
Question 4^a^	40	1.83 (1.92) 0.5 (0–4)	17	1.53 (1.63) 1 (0–3)	0.30	0.58 0.71
Question 5^a^	40	1.35 (1.61) 0.5 (0–3)	17	1.18 (1.59) 0 (0–2)	0.17	0.71 0.76
Question 6^a^	40	1.58 (1.85) 0 (0–4)	17	1.24 (1.68) 0 (0–3)	0.34	0.52 0.57
Question 7^a^	40	1.68 (1.83) 1 (0–4)	17	1.47 (1.66) 1 (0–3)	0.20	0.69 0.91
Question 7 >0^b^	40	21 (53)	17	10 (59)	−6%	0.66
Question 8^a^	40	0.93 (1.47) 0 (0–1.5)	17	0.77 (1.30) 0 (0–1)	0.16	0.70 0.92
Question 9^a^	40	0.75 (1.41) 0 (0–1)	17	0.35 (1.06) 0 (0–0)	0.40	0.30 0.28
Question 10^a^	40	2.05 (1.66) 2 (0–4)	17	2.35 (1.90) 4 (0–4)	−0.30	0.55 0.48
AUDIT-C score	40	4.95 (4.87) 5.5 (0–10)	17	5.53 (4.26) 6 (2–9)	−0.58	0.67 0.63
AUDIT-C ≥3 (F), ≥4 (M)^b^	40	21 (54)	17	11 (65)	−12%	0.40
AUDIT-D score^c^	40	4.75 (5.10) 3 (0–10.5)	17	3.94 (4.26) 3 (0–8)	0.81	0.57 0.69
AUDIT-H score^c^	40	5.40 (4.54) 4 (0–8.5)	17	4.94 (4.35) 5 (1–8)	0.46	0.73 0.77
Total AUDIT score^c^	40	15.10 (13.68) 13.5 (2–27.5)	17	14.41 (11.90) 14 (4–27)	0.69	0.86 0.96

a *p* values are from a *t* test and ordinal logistic regression.

b *p* value is from a chi-square test.

c *p* values are from a *t* test and a Wilcoxon rank sum test.

Abbreviations: AUDIT, Alcohol Use Disorder Identification Test; AUDIT-C, Alcohol Use Disorder Identification Test-Consumption; AUDIT-D, Alcohol Use Disorder Identification Test-Dependence; AUDIT-H, Alcohol Use Disorder Identification Test-Harm.

Patients evaluated by hepatology had lower rates of nonzero answers to question 7 (AUDQ7), regarding guilt and drinking, with 32/87 (37%) of the patients evaluated by hepatology compared to 26/44 (59%) of controls (*p* = 0.02) having nonzero answers, with mean scores also lower but not statistically significant (Table 2).

### AUDIT week 52

There were no significant differences in AUDIT scores between patients seen by hepatology and controls at 52 weeks (Table 2). Similarly, in the patients evaluated by hepatology, there was no significant difference in either mean AUDIT scores or frequency of return to alcohol use in those with evidence of liver disease and those without liver disease (Table 2).

### Hepatology subgroup

All 87 patients in the hepatology group had VCTE data. Of these, 75 had week 1 VCTE, with a mean stiffness of 7.9 (SD: 10.7), 81 with week 2 with mean stiffness of 7.3 (SD: 8.8), and 76 patients with week 4 VCTE, with mean LSM 7.5 kPa (SD: 9.4) (Table 1). At week 1, 21/75 (28%) had LSM ≥7 kPa, compared to 19/81 (23%) at week 2 and 16/76 (21%) at week 4.

When compared to the group with normal liver stiffness, the group with elevated LSM drank significantly more drinks per day (mean: 18.7 vs. 14.3, *p* = 0.02) and began drinking at an earlier age (mean: 11.4 vs. 15.3, *p* = 0.02). The group with elevated LSM also had more heavy-drinking years (median: 21.9 vs. 12.7 y, *p* = 0.05) and more total lifetime drinks (median: 1221 vs. 612 kg, *p* = 0.007) compared to those with normal liver stiffness. Patients with elevated LSM had significantly increased median GGT, platelet count, alanine aminotransaminase, and aspartate aminotransferase that persisted from day 1 to day 23 compared to controls (Table 3). There was no difference in baseline AUDIT score or in rates of prescribed pharmacotherapy (*p* = 0.06). There were also no significant differences in current or lifetime mental health diagnoses (Table 3). There were no significant differences between the AUDIT scores at either week 26 or week 52 (Table 3).

**TABLE 3:** Hepatology subgroup, week 4 kPa <7 versus ≥7

	Liver kPA <7		Liver kPA ≥7		
	N	Mean (SD) Median (IQR) N (%)	N	Mean (SD) Median (IQR) N (%)	*p*
Year of treatment^a^	60	2020.0 (1.14)	16	2019.8 (1.11)	0.59
Before April 2020^b^	60	33 (55)	16	9 (56)	0.93
Sex, male^b^	60	38 (63)	16	13 (81)	0.18
Race, White^b^	60	30 (50)	16	9 (56)	0.66
Age^a^	60	46.6 (12.0)	16	47.2 (8.4)	0.86
Frequencies of alcohol consumption					
Age at first drink^c^	60	15.3 (4.2) 16 (14–17)	16	11.4 (4.7) 13 (8–15)	0.0018 0.0054
Average drink per day^c^	60	14.3 (6.7) 12.9 (9.0–17.1)	16	18.7 (7.5) 17.1 (15.0–24.1)3	0.024 0.022
Drinks per 30 d^c^	60	335 (230) 314 (157–472)	16	477 (272) 449 (383–708)	0.039 0.044
Heavy-drinking days in last 90 d^c^	60	66.0 (26.2) 78 (43–90)	16	68.3 (30.6) 86 (59–90)	0.76 0.69
Heavy-drinking years^c^	59	15.2 (10.6) 12.7 (8.0–20.5)	16	20.9 (9.7) 21.9 (12.6–28.5)	0.056 0.045
Number of drinking days in last 90 d^c^	60	66.4 (24.7) 80.5 (48–90)	16	70.4 (27.0) 86 (59–90)	0.78 0.64
Total lifetime number of drinks (thousands)^c^	59	63.9 (60.2) 43.7 (26.1–80.2)	16	98.9 (51.9) 87.2 (62.2–131.3)	0.037 0.0065
Total lifetime drink^c^ (kg)	59	895 (842) 612 (366–1122)	16	1385 (726) 1221 (870–1838)	0.037 0.0065
Day 1 lab values					
Total bilirubin^a^	60	0.58 (0.32) 0.50 (0.35–0.70	16	1.13 (2.0) 0.65 (0.35–0.95)	0.29 0.34
GGT^c^	60	126 (151) 66 (33–145)	16	430 (698) 181 (92–415)	0.10 0.0026
MCV^a^	60	93.7 (5.71) 93.8 (91.3–97.0)	16	95.9 (6.96) 95.7 (91.7–97.7)	0.20 0.33
Platelet count^a^	59	229 (81.7) 227 (169–294)	16	185 (87.2) 152 (135–249)	0.060 0.044
ALT^c^	60	45.4 (38.9) 34 (24–59)	16	55.1 (27.7) 53 (31–83)	0.36 0.080
AST^c^	60	55.1 (42.4) 38 (22–77)	44	102.9 (75.4) 87 (45–142)	0.026 0.011
Day 21 lab values					
Total bilirubin^a^	59	0.37 (0.17) 0.30 (0.20–0.50)	16	0.82 (1.74) 0.30 (0.25–0.5)	0.32 0.81
GGT^c^	59	60.1 (61.3) 43 (26–68)	16	140.7 (158.2) 76 (63–135)	0.063 0.0009
MCV^a^	52	93.4 (5.53) 93.2 (89.6–97.6)	16	96.2 (5.71) 95.5 (92.8–98.7)	0.086 0.16
Platelet count^a^	52	274 (74.3) 271 (219–316)	16	223 (87.4) 220 (156–278)	0.034 0.057
ALT^c^	59	26.0 (12.3) 23 (18–30)	16	39.6 (21) 29 (24–55)	0.025 0.014
AST^c^	59	23.2 (7.9) 21 (17–27)	16	38.2 (24.0) 29 (26–37)	0.026 0.0006
Baseline AUDIT					
Question 1^d^	60	3.80 (0.58) 4 (4–4)	16	3.81 (0.75) 4 (4–4)	0.94 0.48
Question 2^d^	60	3.40 (0.94) 4 (3–4)	16	3.25 (1.13) 4 (2.5–4)	0.59 0.71
Question 3^d^	60	3.55 (0.72) 4 (3–4)	16	3.50 (1.10) 4 (3.5–4)	0.86 0.63
Question 4^d^	60	3.18 (1.21) 4 (3–4)	16	3.00 (1.37) 3.5 (2.5–4)	0.64 0.57
Question 5^d^	60	2.08 (1.25) 2 (1–3)	16	1.94 (1.29) 2 (1–3)	0.68 0.66
Question 6^d^	60	2.35 (1.55) 3 (1–4)	16	2.63 (1.75) 3.5 (0.5–4)	0.54 0.43
Question 7^d^	60	2.52 (1.31) 3 (1–4)	16	2.75 (1.48) 3 (1.5–4)	0.54 0.37
Question 8^d^	60	1.87 (1.24) 2 (1–3)	16	1.50 (1.37) 1 (0–3)	0.31 0.29
Question 9^d^	60	1.23 (1.69) 0 (0–2)	16	0.88 (1.63) 0 (0–1)	0.45 0.37
Question 10^d^	60	3.57 (1.17) 4 (4–4)	16	3.50 (1.16) 4 (4–4)	0.84 0.65
AUDIT-C score^c^	60	10.8 (1.8) 12 (10–12)	16	10.6 (2.1) 12 (9.5–12)	0.73 0.98
AUDIT-D score^c^	60	7.6 (3.1) 8 (6–11)	16	7.6 (3.1) 8.5 (5.5–9.5)	0.95 0.98
AUDIT-H score^c^	60	9.2 (3.6) 10 (7–12)	16	8.6 (3.2) 9 (7–11)	0.57 0.56
AUDIT total score^c^	60	27.6 (6.8) 28 (22–34)	16	26.8 (5.9) 28 (26.5–29.5)	0.67 0.57
Other SUD current^b^	60	18 (30)	16	3 (19)	0.37
Other SUD lifetime^b^	60	38 (63)	16	10 (63)	0.95
Anxiety current^b^	60	21 (35)	16	4 (25)	0.45
Anxiety lifetime^b^	60	31 (52)	16	8 (50)	0.91
Anxiety, no PTSD or OCD current^b^	60	12 (20)	16	2 (13)	0.49
Anxiety, no PTSD or OCD lifetime^b^	60	14 (23)	16	3 (19)	0.70
Mood current^b^	60	17 (23)	16	1 (6)	0.082
Mood lifetime^b^	60	26 (43)	16	4 (25)	0.18
Depression current^b^	60	15 (25)	16	1 (6)	0.10
Depression lifetime^b^	60	25 (42)	16	4 (25)	0.22
PTSD current^b^	60	11 (18)	16	3 (19)	0.97
PTSD lifetime^b^	60	20 (33)	16	6 (38)	0.75
Pharmacotherapy for AUD					
Prescribed at discharge	60	38 (63)	16	6 (38)	0.063
LSM liver kPA					
LSM week 1 (kPa)^c^	50	5.5 (2.9) 5.1 (3.8–6.2)	14	18.9 (22.0) 12.1 (7.0–14.8)	0.040 <0.0001
LSM week 2 (kPa)^c^	55	4.8 (2.0) 4.5 (3.6–5.7)	16	17.2 (16.6) 11.4 (7.4–16.8	0.0091 <0.0001
LSM week 4 (kPa)	60	4.5 (1.0) 4.6 (3.8–5.2)	16	18.5 (16.6) 12.1 (9.0–20.5)	NA
Week 26 Audit					
Question 1^d^	60	1.37 (1.72) 0 (0–3)	16	1.56 (1.97) 0 (0–4)	0.66 0.65
Question 1 >0^b^	60	26 (43)	16	7 (44)	0.98
Question 7 >0^b^	60	20 (33)	16	6 (38)	0.75
AUDIT-C ≥3 (F), ≥4 (M)^b^	60	25 (42)	16	6 (38)	0.76
Week 52 Audit					
Question 1^d^	16	1.57 (1.77) 1 (0–4)	7	2.14 (1.77) 3 (0–4)	0.45 0.51
Question 1>0^b^	28	15 (54)	7	5 (71)	0.39
Question 7 >0^b^	28	13 (46)	7	5 (71)	0.24
AUDIT-C ≥3 (F), ≥4 (M)^b^	28	13 (46)	7	5 (71)	0.24

a *p* value is from a *t* test.

b *p* value is from a chi-square test.

c *p* values are from a *t* test and a Wilcoxon rank sum test.

d *p* values are from a *t* test and ordinal logistic regression.

Abbreviations: ALT, alanine aminotransaminase; AST, aspartate aminotransferase; AUD, alcohol use disorder; AUDIT, Alcohol Use Disorder Identification Test; AUDIT-C, Alcohol Use Disorder Identification Test-Consumption; AUDIT-D, Alcohol Use Disorder Identification Test-Dependence; AUDIT-H, Alcohol Use Disorder Identification Test-Harm; GGT, gamma-glutamyl transferase; LSM, liver stiffness measurement; MCV, mean corpuscular volume; OCD, obsessive-compulsive disorder; PTSD, post-traumatic stress disorder; SUD, substance use disorder.

### Control subgroup

The AUDIT scores of patients with evidence of abnormal labs at week 3 based on laboratory reference criteria were compared with patients with normal labs. There was no difference in AUDIT scores seen at either week 26 or week 52 (Table 4).

**TABLE 4:** Control subgroup by elevated transaminases by reference value at week 3

	Normal^a^ AST and ALT		ALT ≥56 or AST ≥5			
Variable	N	N (%) Percent	N	N (%) Percent	Diff (%)	Exact *p*
Week 26						
AUDIT Q1 >0	17	11 (65)	9	7 (78)	13	0.67
AUDIT Q7 >0	17	10 (58)	9	4 (44)	−14	0.68
AUDIT-C ≥3 (F), ≥4 (M)	17	10 (59)	9	6 (67)	8	1.00
Week 52						
AUDIT Q1 >0	6	5 (83)	4	2 (50)	−33	0.50
AUDIT Q7 >0	6	4 (67)	4	1 (25)	−42	0.52
AUDIT-C ≥3 (F), ≥4 (M)	6	5 (83)	4	1 (25)	−58	0.19

a Normal as defined by a reference laboratory.

Abbreviations: ALT, alanine aminotransaminase; AST, aspartate aminotransferase; AUDIT, Alcohol Use Disorder Identification Test; AUDIT-C, Alcohol Use Disorder Identification Test-Consumption.

## DISCUSSION

Our study suggests that the integration of a hepatologist in inpatient alcohol cessation treatment reduces rates of early return to alcohol use and identifies patients with ALD. Patients evaluated by hepatology during inpatient treatment of AUD had significantly lower rates of return to alcohol use at 26 weeks than patients who did not have hepatology evaluation. Patients evaluated by hepatology also had lower rates of hazardous drinking defined by AUDIT-C at 26 weeks. These findings suggest that hepatology evaluation during inpatient treatment of AUD may influence short-term behavior and ultimately lead to decreased rates of early return to alcohol use. This response was not durable, with a difference in return to drinking only seen at 26 weeks, but no difference seen at 52 weeks.

Up to 70% of the patients who undergo treatment for AUD return to alcohol use within the first 12 months, with the highest percentage returning to drinking within 3 months. Our overall rate of return to alcohol use was 41% at 6 months, with 65% of the patients returning to drinking at 12 months. This is likely an underestimation, as 45% of the patients who enrolled in the inpatient treatment program were lost to follow-up.

Our intervention and control group were nearly identical, except for increased diagnoses of anxiety and increased alcohol consumption in the group seen by hepatology. A possible explanation for both these differences is that most of the patients in the control group were seen before the COVID-19 pandemic. It has been well documented in multiple studies that there was an increase in alcohol use with an acute rise in ALD during the pandemic.^21^ Similarly, the prevalence of anxiety diagnoses increased during the pandemic, possibly accounting for some of these differences.^22^

### Why does hepatology consultation help?

We theorized that patients with evidence and knowledge of ALD would demonstrate a behavior change with lower rates of return to drinking.

### AUD interventions

Treatment of AUD often requires a multimodal approach with a combination of behavior therapy and pharmacotherapy.^23^ Multiple studies show that medications for AUD are effective and are associated with decreased incidence and progression of liver disease.^9^,^10^ In our study, patients seen by hepatology participated in the same inpatient alcohol treatment program and had similar rates of prescription for pharmacotherapy at discharge to controls, suggesting that an effect beyond behavioral and medical therapy occurred during the hepatology consults leading to decreased return to alcohol use at 26 weeks.

The hepatology consultation consisted of a discussion of laboratory results, including liver-associated enzymes, complete blood count, and inflammatory markers, and a fibroscan with a discussion of the results. All patients were counseled on complete abstinence from alcohol regardless of the individual results and received education on the deleterious effects of alcohol on the liver. This brief but targeted intervention in combination with multimodal treatment for AUD likely increased motivation for alcohol cessation in the patients evaluated by hepatology, with some evidence that brief interventions outside of addiction care are associated with decreased alcohol consumption.^24^

Our results differ from Mahle and colleagues, where inpatients hospitalized with AUD were evaluated by hepatology. In this study, there was no measurable difference in early remission or partial remission between groups who saw hepatology and those who did not. Notably, this study did not specify whether the inpatient population was treatment-seeking. One possible explanation for this discrepancy is differences in the treatment population as well as the number of times that patients in our study saw a hepatologist for evaluation of the liver.^25^

### Knowledge of liver stiffness

While patients with elevated LSM had significantly higher rates of alcohol use before admission including average drinks per day and total lifetime drinks, there were no differences in AUDIT scores between patients with normal and elevated LSM. Reassuringly, patients with normal LSM did not have higher rates of return to alcohol use when compared to those with elevated LSM. These results suggest that the knowledge that the liver did not sustain lasting injury despite heavy alcohol use did not provide false reassurance.

Our results are consistent with the results of prior studies, including a randomized controlled trial that leveraged VCTE and video interventions in a community-based alcohol intervention program. This study found at 6-month follow-up that the intervention group had a greater reduction in alcohol intake and was more likely to complete the program. Of the 52 patients who received VCTE, none of the patients reported an increase in alcohol intake or AUDIT category, suggesting that normal LSM did not provide false reassurance. This study demonstrated a trend toward longer duration of services in the intervention group when compared to the control. However, this study was underpowered to show statistical differences in key indicators of behavior change.^14^

Our results differ from a 2008 study on the influence of liver biopsy on abstinence in patients with AUD. This study suggested that patients with more severe liver disease on biopsy had a lower rate of rapid return to alcohol use, though long-term abstinence was similar in all histopathology groups.^12^ This study included 137 patients who underwent liver biopsy, with 28 patients identified as having severe liver disease. The patients who were categorized as severe had lower rates of return to drinking at 3 months, but not at 12 months. Overall, patients who had liver biopsies trended toward higher rates of early return to alcohol use with significantly worse rates of long-term abstinence than controls, though patients who had liver biopsies were generally sicker.

### Detection of ALD

While our study aimed to examine the association between hepatology consultation and rates of return to alcohol use, we found that a significant portion of our heavy-drinking study population had evidence of liver disease, with 21% having LSM ≥7 kPa at week 4. This finding is consistent with other studies, where 18%–27% of the populations have significant risk factors for liver disease.^26^

Given that the patients with ALD are often diagnosed at the late stages of the disease and abstinence is key to preventing the progression of ALD, integration of hepatology services into the treatment of patients with AUD is under study for feasibility and outcomes. In one study where hepatologists evaluated patients admitted with AUD and performed VCTE, advanced liver disease was diagnosed in 30% of consulted patients, based on week 4 elastography with LSM ≥13 kPa or on liver biopsy.^27^

### Contextual factors

These data must be interpreted in the context of the study design. First, all patients participated in the same inpatient multidisciplinary treatment program and received labs, imaging, and therapy, minimizing confounding factors. Furthermore, to our knowledge, this is the first study where hepatology was integrated into an inpatient treatment program for AUD. It also adds to the growing body of literature that personalized biomarker-based advice can enhance motivation to overcome addictive behaviors.^28^

Second, this was a nonrandomized study with our control group taken from convenience sampling. Nonetheless, the intervention group and the control group were similar populations who underwent identical inpatient treatment, outside of the hepatology consultation. This study is unable to account for social support or socioeconomic factors that may affect a return to alcohol use. The study population only included patients with AUD who were treatment-seeking. It also could not account for inherent selection bias in patients who responded to AUDIT (loss of follow-up bias). In our study, we used AUDIT-C as a surrogate for return to alcohol use. As such, there was some missing data from some questions, with some patients selectively answering the AUDIT questions. AUDIT is also not routinely used to measure the return to alcohol use. Another major limitation of this study is that we did not directly assess patient-reported drinking or use objective biomarkers such as serum phosphatidylethanol or urine ethyl glucuronide. Furthermore, as fellows used their clinical judgment to counsel patients rather than a script, this is a source of variability. Also, there was no way to discern what component of the hepatology consult (education, VCTE, and discussion of lab values) was most effective.

## CONCLUSIONS

Integration of a hepatologist in inpatient alcohol treatment programs may improve rates of early return to alcohol use and can detect early ALD. Furthermore, alcohol treatment programs are an ideal opportunity for early diagnosis of ALD and provide an opportunity for early intervention. Future studies should explore which specific component of the hepatology consult had the greatest impact on behavior change, as well as whether longitudinal follow-up with hepatology reduces rates of return to alcohol use.

## Data Availability

Data, analytic methods, and study materials will not be shared.
